# The Influence of an Attachment-Related Stimulus on Oxytocin Reactivity in Poly-Drug Users Undergoing Maintenance Therapy Compared to Healthy Controls

**DOI:** 10.3389/fpsyt.2020.460506

**Published:** 2020-09-25

**Authors:** Jürgen Fuchshuber, Jasmin Tatzer, Michaela Hiebler-Ragger, Florian Trinkl, Andreas Kimmerle, Anita Rinner, Anna Buchheim, Silke Schrom, Beate Rinner, Klaus Leber, Thomas Pieber, Elisabeth Weiss, Andrew J. Lewis, Hans-Peter Kapfhammer, Human Friedrich Unterrainer

**Affiliations:** ^1^Department of Psychiatry and Psychotherapeutic Medicine, Medical University Graz, Graz, Austria; ^2^CIAR: Center for Integrative Addiction Research, Grüner Kreis Society, Vienna, Austria; ^3^Institute of Psychology, University of Graz, Graz, Austria; ^4^Department of Clinical Psychology, Institute of Psychology, University of Innsbruck, Innsbruck, Austria; ^5^Department of Biomedical Research, Medical University Graz, Graz, Austria; ^6^Department of Neurosurgery, Medical University Graz, Graz, Austria; ^7^Department of Endocrinology and Diabetology, Medical University of Graz, Graz, Austria; ^8^Discipline of Psychology, College of Science, Health, Engineering & Education, Murdoch University, Perth, WA, Australia; ^9^Department of Religious Studies, University of Vienna, Vienna, Austria

**Keywords:** attachment, maintenance treatment, poly drug use, oxytocin, substance use disorder

## Abstract

**Background:**

Substance use disorders (SUDs) have been described as a dysfunctional way to compensate for deficiencies in that person’s underlying attachment system. Furthermore, the neuropeptide oxytocin (OT), which is a critical component of the neurobiology of the attachment system, has been shown to effectively reduce addictive behavior and therefore has been discussed as a potential medication in SUD treatment. This study investigates variation in peripheral OT plasma levels as a function of exposure to an attachment-related stimulus in SUD patients compared to healthy controls (HCs).

**Methods:**

A total sample of 48 men, 24 inpatients in maintenance treatment who were diagnosed with poly-drug use disorder (PUD) and 24 HC, was investigated. A 15-min exposure to the Adult Attachment Projective Picture System (AAP) was used as an attachment-related stimulus and coded for attachment status. Blood samples before and after the AAP-assessment were taken and assayed for OT levels. Variation in baselines level of OT was examined in relation to the Alcohol, Smoking and Substance Involvement Screening Test (ASSIST), the Adult Attachment-Scale (AAS), and the Brief Symptom Inventory (BSI).

**Results:**

Following the AAP stimulus controls showed no significant difference in OT levels elevation from baseline compared to the PUD group’s OT levels. Furthermore, in the PUD group only OT-baseline-levels may be negatively associated with the AAS subscale “Comfort with Closeness” and “Anxiety” and lifetime substance use.

**Discussion:**

Our results suggest that peripheral OT levels in poly-drug users undergoing maintenance treatment are not significantly different in responsiveness to an attachment related stimulus compared to HC. With regard to non-significant tendencies observed in this study which hint toward decreased OT-reactivity in the PUD group, further research is needed to explore this hypothesis with increased statistical power.

## Introduction

Substance use disorders (SUDs) have been characterized as a compulsive substance use without consideration of the negative consequences (1) and are increasingly framed as a neurobiological disorder (2, 3). Currently, the most common form of SUD in patients undergoing treatment in Austria is poly-drug use disorder (PUD), with opioids as the primary drug of choice (4), a pattern which is also found in the majority of SUD patients across Europe (5). In recent years, increasing number of patients are treated within maintenance treatment programmes, which have been shown to be effective treatments by reducing heroin use and risk behaviors as well as improving health, social and criminal justice outcomes (6).

From a psychodynamic perspective, SUD has been understood in relation to attachment disorder (7) and as a dysfunctional way of self-medicating (8). Specifically, insecure attachment has been linked to increased psychopathology for decades (9). Formed by early parent-infant interactions, which are gradually imprinted in neuronal pathways (10, 11), attachment can be understood as a neurobiological system designed to promote social affiliation and primary bonding experiences (12, 13). Recent studies indicate a substantial role of insecure attachment in the etiology of SUDs (14–16)—among other psychiatric disorders (17). This relationship has been linked to the influence of attachment styles on the interpersonal regulation of human emotions particularly fear, anxiety and hedonic experiences within close relationships (18, 19).

Attachment research across mammalian species has suggested that the neuropeptide oxytocin (OT) plays a central role in the neurobiological processes involved in the formation and maintenance of social bonds (20), interpersonal affect regulation (14, 21) and parent-child relationships (22–24), but also protective aggression (25). The OT-system in humans is associated with brain regions including the amygdala, paraventricular nucleus (PVN), supraoptic nucleus (SON), ventral pallidum (VP), ventromedial nucleus of the hypothalamus (VMH), area tegmentalis ventralis (VTA), substantia nigra (SN), and the neuroendocrine systems (26). Consisting of nine amino acids, this neuropeptide is produced by PVN and SON. Through axonal transport OT is centrally released to hippocampus, amygdala, striatum, hypothalamus, nucleus accumbens, and the central brain in response to social interactions and stressors (27–30). In line with the Calm and Connect Model (31), which assumes that bonding, experienced through touch and social affection, leads to OT production and thus positively reinforces social connection, several studies have linked insecure attachment patterns to impairments of the OT-system (23, 27, 29, 32).

In the context of addiction, beneficial effects of administered OT on drug tolerance, withdrawal and seeking have been proposed across various substance classes (33, 34). Individual differences in the endogenous OT-system may therefore affect the vulnerability to addiction. SUDs have been repeatedly linked to decreased levels of OT (35–37). Furthermore, OT is assumed to modulate the mesolimbic dopamine system (38), a structure which is substantially involved with the process of addiction development and bond formation (2, 39). Similarly, there is considerable evidence suggesting interactions between the OT and endogenous opioid system (40). In line with these observations, a recent review by Zanos et al. (41) concluded that the OT system is not only meaningfully influenced by opioid addiction and abstinence but also might serve as a critical target for pharmacological interventions. Such findings inform the first aim of this study to investigate cross sectional relationships between substance use and OT levels.

Previous research indicated a relationship between the administration of stimuli designed to activate the attachment system of participants and the OT-system. One such measure, the Adult Attachment Projective Picture System (AAP) was shown to significantly increase OT levels (42). This study was conducted with a sample of healthy lactating mothers who might be thought to be especially responsive to attachment cues. Moreover, these authors hypothesized that women with more secure attachment patterns should show higher OT-reactivity. However, in this study, the authors were not able to confirm the proposed association between a larger increase in OT and more securely attached mothers. This experimental paradigm using the AAP as an attachment stimulus is adopted in the current study, while our study is focused on substance users compared to healthy controls (HCs).

What is more, in recent years, several reviews have been published which critically asses methodical flaws frequently observed within the research of the human OT system [e.g., (43–45)]. These contributions specifically emphasize the importance targeted hypotheses, consideration of differences between central processing of OT and its peripheral levels, as well studies focussed on peripheral levels making use of plasma samples, and plasma to be assayed for OT levels after extraction.

With this in mind, this study aimed to enhance the understanding the relationship between attachment and the OT-system in patients with SUD. We sought to address two primary aims. First, using baseline levels of peripheral OT, we examined their associations with substance use (using the ASSIST), attachment (using the Adult Attachment-Scale), and current symptoms (using the Brief Symptom Inventory). In relation to the first aim, we expected to find OT levels negatively associated with insecure attachment patterns and psychopathological symptom burden in the PUD group. Our second aim follows the experimental study by Krause et al. (42), which focuses on the response of the peripheral OT-system in response to an attachment-related stimulus. In the experimental study, we compared PUD patients undergoing maintenance therapy to HCs. Following Krause, we expected to see a rise in the OT levels of health controls when exposed to an attachment stimulus. We were exploring whether the SUD group would show a different OT response to the same stimulus. However, as this is the first time, this experimental paradigm is investigated in patients undergoing maintenance treatment, this hypothesis remains exploratory.

## Sample and Methods

### Participants

The study sample consisted of 48 male participants between 19 to 38 years of age (*M* = 27.42, *SD* = 4.82), consisting of one clinical (PUD; *n* = 24) and one non-clinical group (HC; *n* = 24). Participants in the clinical group met diagnostic criteria for PUD (F19.2), diagnosed according to the International Classification of Diseases version 10 (ICD 10) (46) by a licensed psychiatrist. Due to the haphazard drug use, one of the main characteristics for PUD, the drugs consumed cannot be reported in detail. At the time of the study, all PUDs were currently participating in maintenance therapy as described below. PUDs with fluid psychotic symptoms were excluded. Comorbidities with other diagnoses were distributed as follows: 9.2% Affective disorders (F3.x), 5.8% Neurotic, stress and somatoform disorders (F4.x), 4.6% Personality and behavioral disorders (F6.x), 2.3% Schizophrenia, schizotypal and delusional disorders (F2.x), 1.2% Behavioral and emotional disorders (F5.x) with onset usually occurring in childhood and adolescence.

Before participating in the study PUD patients had been in maintenance therapy for a mean time of 15 weeks (*SD* = 13.8) and received either *Levo-Methasan (n* = 21*)*, *Bupensan (n* = 1*)*, *Substitol Retard* (*n* = 1), or *Compensan Retard* (*n* = 1) as a substitution agent, with daily doses ranging from 2 to 320 mg, depending on patient and medication. Furthermore, 21 PUD patients received additional psychopharmacological medication: 16 (66.67%) received antipsychotics and 19 (79.17%) received antidepressants. Participants of the non-clinical group, exclusively non-smoking men, reported either none or just a few previous experiences with illegal substances. With the exception of occasional consumption of alcohol, no use of psychoactive substances was reported by HC in the last 30 days prior to the investigation and no use of psychopharmacological medication. HCs were included if they reported no past or present psychiatric disorder or chronic disease.

Exclusion criteria for both groups were insufficient knowledge of the German language. Clinical subjects were assessed at the Johnsdorf therapeutic facility of the Grüner Kreis Society. Non-clinical subjects were recruited through advertising on social networks and *via* email distribution of the University of Graz. The study was approved by the ethics committee of the University of Graz, Austria and conducted in accordance with the Declaration of Helsinki.

### Procedure and Design

In order to eliminate any effects due to circadian rhythms the timing of the experiment was standardized. Participants were asked to fast for at least 3 hours before arriving in the laboratory (between 12.00 am and 3.30 pm), avoid caffeinated drinks and to refrain from smoking on the day of participation, before and during the experiment. After written informed consent was obtained and the subjects were notified about the course of the experiment, the first venipuncture and blood collection was performed. Immediately after, the AAP (47) was applied in which participants were asked to tell a story for each of the eight shown pictures with either monadic or dyadic scenes by answering the following questions: “What is happening in the scene?”, “What led up to the scene?”, “What are the characters thinking or feeling?”, and “What might happen next?”. The abstract line drawings indicate scenarios such as illness, separation, and abuse without detailed facial expression, allow a large scope of interpretation (47). The AAP measure is designed around a common assumption in observational and discourse attachment measures that attachment behavior is best observed directly after an attachment related stimulus is delivered or represented such as a separation, loss, illness and so on (48). The interviews lasted on average 16 min (*SD* = 4.50). The AAP interviews were administered by a trained psychologist in a standardized manner according to the published administration requirements. Following the AAP, and 25 min after the first blood sample a second blood sample was collected, again *via* venipuncture. The psychometric assessment (described below) took place online *via* Lime-Survey^®^ before the experiment.

### Measures

#### Addictive Behavior

The German Version of the *Alcohol*, *Smoking and Substance Involvement Screening Test* [*ASSIST 3.0*; (49), German Version; (50)] is a structured short interview designed to record life-time consumption behavior and its negative effects from the following substance classes: alcohol, tobacco, cannabis, cocaine, amphetamines, inhalants, sedatives, hallucinogens, and opiates among others. For this study, the interview was adapted as a self-report questionnaire. Questions about the “Frequency of drug use”, “Craving to use the drug”, “Problems”, and “Failed expectations” are rated on a 7-point Likert scale from 0 (never) to 6 (daily). Questions about “Expressed concerns by relatives or friends”, “Failed attempts to cut down drug use”, and “Drug injection” are rated on a 3-point Likert scale (0 = “no never”, 3 = “yes, but not in the past 3 months”, 6 = “yes, in the past 3 months”). By adding the drug specific symptom scores an overall score for every symptom class (mentioned above), as well as a total score was calculated. Subscales ranged in Cronbach’s alpha from 0.79 to 0.89.

#### Mental Health Symptoms

The short version of the *Brief Symptom Inventory* [*BSI-18*; (51), German Version: (52)] assesses the amount of psychiatric burden of the last 7 days by means of 6 items on each of the three subscales: (1) Somatization, (2) Depression, and (3) Anxiety. It is rated on a 5-point Likert scale from 0 “absolutely not” to 4 “very strong”. A Global Severity Index (*GSI*) can be generated for a total of the 18 items. Cronbach’s alpha for the subscales ranged from 0.70 to 0.87. The total Global Severity Index score showed a Cronbach’s alpha of 0.87.

#### Attachment Styles

The German Version of the *Adult Attachment Scale* [*AAS*; (53, 54)] is a self-report method measuring attachment dimensions based on attachment theory (55). This questionnaire consists of three subscales: (1) Anxiety about being rejected or unloved, (2) Comfort with Closeness and Intimacy, and (3) Comfort in Depending on others. This questionnaire consists of 18 items rated on a 5-point Likert scale ranging from 1 (strongly disagree) to 5 (strongly agree). Cronbach’s alpha for the scales ranged from 0.68 for to 0.79.

#### Oxytocin Assessment

For measuring the plasma OT levels, blood samples were drawn from antecubital veins into 3-ml vacutainer blood vacuettes (Greiner Bio-One International GmbH, Austria) containing Aprotinin (500 KIU/ml of blood) (Sigma-Aldrich, Germany). Vacuettes were stored at −20°C before use. Vacuettes were centrifuged at 4°C at 1.600 g for 15 min. Supernatants were stored at −80°C until analysis. Extraction of samples was undertaken and OT concentrations in the extracts were determined in duplicate by *Oxytocin ELISA kit* (ADI-900-153A, Enzo Life Sciences, USA), a colorimetric competitive enzyme immunoassay kit at the Center for Medical Research at the Medical University Graz, Austria. The mean intra-assay and inter-assay coefficients of variability were 23.4% and 13.9%, respectively; sensitivity was 15.0pg/ml. All procedures were performed according to the manufacturer’s instructions by authorized personnel.

### Data Reduction and Statistical Analyses

For group comparisons in the experimental design, one-way analyses of variance and χ² tests were conducted. To evaluate the reactivity of OT, the amount of the difference value of pre- and post-OT-level was considered. To investigate the relationship between OT and behavioral measures Pearson’s correlation coefficients were calculated separated for the PUD group. Alpha was set to *p* < 0.05 in ANOVAs and Pearson’s correlations. However, with regard to recent critical reviews of OT-literature [e.g., (43, 44)], we additionally corrected for multiple comparisons *via* the Bonferroni correction. In order to ensure a better evaluation of the results, effect sizes were included.

## Results

### Demographics and Clinical Characteristics

Socio-demographic variables, scores for addictive behavior as well as requirements prior to the interview of both groups are presented in **Table 1**.

**Table 1:** Group differences in demographic data and conditions prior to investigation.

	PUD (*n* = 24)		HC (*n* = 24)		*T*	*df*	*p*
	*M*	*SD*	*M*	*SD*			
Age	28.50	5.85	26.33	3.25	-1.59	35.99	0.119
Risk of substance use							
Lifetime substance use (incl. alcohol & tobacco)	23.63	4.79	8.63	4.18	-11.56*	45.17	0.000
Global continuum of substance risk (incl. alcohol & tobacco)	29.04	4.43	13.75	7.04	-9.01	46	0.000
Conditions day of examination							
Waking up	467.17	79.61	371.96	138.38	-2.92*	46	0.005
Caffeine consumption^a^	440.63	178.57	–	–	–	–	–
Nicotine consumption^a^	103.54	195.23	–	–	–	–	–
Last meal^a^	272.63	133.84	360.04	256.75	1.48	34.64	0.146
Sexual activity	700.43	138.25	621.25	231.45	-1.43	37.83	0.161
	**PUD (*n* = 24)**		**HC (*n* = 24)**		***X²***	***df***	***p***
Nationality	***n***		***N***		7.54	4	0.110
Austria Other Country	16 8		19 5				
German language skills					4.73	2	0.094
Mother tongue Very well Less well	16 7 1		22 2 0				
Education					48.00*	5	0.000
No completed Education Secondary school Apprenticeship High School Bachelor Master/Doctor	1 10 12 1 0 0		0 0 0 14 5 5				
Psychiatric diagnosis					45.15*	1	0.000
Yes	24		0				
Current psychotherapy					49.00*	1	0.000
Yes	24		0				
Chronic physical health problems					3.33	1	0.068
Yes	3		0				
Regular medication					49.00*	1	0.020
Yes	24		0				

*p < 0.05; PUD, Poly-drug use disordered patients; HC, Healthy controls. ^a^Past time in minutes since last consumption on test day.

### Hypothesis-Testing Results

#### Group Differences in OT and Attachment

As depicted in **Table 2**, group comparisons showed that PUD had higher levels of OT compared to HC before at baseline (*F_(1, 46)_* = 7.02; *p* < 0.05). No other significant group differences regarding OT were observed (all *p* > 0.05) [for comparative means see (56)]. Following the administration of the AAP as attachment stimuli, the HC seemed to increase in OT levels whereas the PUD group’s OT remained flat. However, this difference was not significant (*F_(_* ***_1_*, *_46_****_)_* = 3.25; *p* = 0.08).

**Table 2:** Group differences (ANOVA) in behavioral and biological measures.

Measures	*a*	PUD (*n* = 24)		HC (*n* = 24)		*F*_(_*_1_*, *_46_*_)_	*η²*	*p*
		*M*	*SD*	*M*	*SD*			
BSI-18								
Somatization	0.690	2.17	2.73	2.13	2.35	0.00	0.00	0.955
Depression	0.852	6.25	5.57	2.71	2.33	8.27*	0.15	0.006
Anxiety	0.816	4.54	5.01	3.46	2.41	0.91	0.02	0.344
Total Score	0.869	12.96	11.14	8.71	5.39	2.83	0.06	0.099
Oxytocin								
Pre (pg/ml)		60.64	24.87	44.74	15.68	7.02*	0.13	0.011
Post (pg/ml)		60.38	17.25	60.46	38.73	0.00	0.00	0.992
Reactivity		-0.26	17.64	15.72	39.66	3.25	0.06	0.078
AAS								
Dependence	0.731	16.13	4.89	18.42	3.31	3.61	0.07	0.064
Closeness	0.786	11.63	3.93	13.92	4.03	3.97	0.08	0.052
Anxiety	0.678	12.29	3.91	12.29	3.75	0.00	0.00	1.000

Bonferroni corrected p = 0.005; *p < 0.05; PUD, Poly-drug use disordered patients; HC, Healthy controls; Pre, baseline OT-levels; Post, OT-levels after confrontation with attachment related cue.

Furthermore, the between group tests for differences in the measures of mental health and attachment the PUD group showed a tendency toward less Comfort with closeness (*F_(_* ***_1_*,* _46_****_)_* = 3.97; *p* = 0.05) and Comfort with Depending on others (*F_(_* ***_1_*, *_46_****_)_* = 3.61; *p* = 0.06) and higher depressive symptom burden (*F_(_* ***_1_*, *_46_****_)_* = 8.27; p < 0.05). With regard to the Bonferroni corrected alpha level, no group differences remained significant (all *p* > 0.003)

### Intercorrelations of Oxytocin, Attachment, and Personality Characteristics for PUD

Correlations over PUD showed that baseline OT-levels were related to less Comfort with closeness (*r* = −0.41, *p* < 0.05) and lifetime substance use over all substance classes (*r* = −.48, *p* < 0.05). Furthermore, OT-reactivity showed non-significant tendencies with Comfort with closeness (*r* = .34, *p* < 0.10) and Lifetime substance use (*r* = .37; p = 0.07). Moreover, as shown in **Table 3**, insecure attachment patterns were related to Depression (*r* = −.51–.49; all *p* < 0.05). No correlation remained significant if corrected for multiple comparisons (all *p* > 0.003).

**Table 3:** Intercorrelations for behavioral and biological measures for PUD (*n* = 24).

Variable	1	2	3	4	5	6	7	8	9	10	11
1. BSI-18 Somatization		.40	.79**	−.21	−.03	.27	−.11	−.15	.19	.17	.14
2. BSI-18 Depression			.48*	−.04	.01	.06	−.51*	−.46*	.49*	.21	.21
3. BSI-18 Anxiety				−.13	−.08	.10	−.02	−.12	.22	.14	.34
4. OT Pre					.70**	−.72*	.05	−.41	−.37	−.48*	.11
5. OT Post						−.02	.07	−.24	−.33	−.31	−.04
6. OT Reactivity							.00	.34	.20	.37	−.19
7. AAS Dependence								.68**	−.36	−.05	−.20
8. AAS Closeness									−.02	−.04	−.22
9. AAS Anxiety										−.18	.24
10. ASSIST Lifetime SU											−.16
11. ASSIST GC of SR											

N = 24; Bonferroni corrected p = 0.004; **p < .01, *p < .05; Pre, baseline OT-levels; Post, OT-levels after confrontation with attachment related cue; GC, global continuum; SU, substance use; SR, substance risk.

## Discussion

In order to enhance the understanding of the relationship of OT to SUD, we investigated the differences in psychopathology, attachment, and the OT-system between PUD patients undergoing maintenance treatment compared to HC, as well as differences in peripheral OT response to an attachment-related stimulus. Our results suggest that PUD patients were higher OT at baseline compared to a HC group. In response to the attachment stimulus containing the AAP procedure, differences between the PUD and HC groups regarding OT-reactivity remained non-significant. Furthermore, baseline OT-levels showed a significant relationship with decreased Comfort with closeness in PUD patients.

However, these results should be interpreted with caution. In the first instance, the sample size of the study was small and there were numerous significance tests run. Following Nave et al. (44) and McCullough et al. (43), who proposed the necessity for correcting for multiple comparisons, no finding remained significant based on a Bonferroni corrected alpha level. While the Bonferroni correction has been criticized as being overly conservative (57, 58), the findings of this study are tentative and require replication in a larger study.

The finding of increased OT-baseline in the PUD group is in contrast to many other studies (41). The interpretation of this result needs to remain speculative at this point. However, it is conceivable that this finding might be traced back to the characteristics of living in the therapeutic community which is characterized by high social cohesion and an attachment focused treatment approach (59). Furthermore, in contrast to the HC group, PUD participants traveled to the OT measuring in groups, which might have further contributed to inflated OT baseline levels (60). Another possibility would be an influence of the various medications used for maintenance therapy which interact with the opioid system, or indeed the use of antidepressant or antipsychotic medications in PUD participants. However, while not extensively researched, recent literature indicates no influence of antidepressant pharmacological treatments on OT (61) but there have been some animal studies suggesting a relationship between antidepressants and OT metabolism (62).

OT-reactivity in PUD patients did not significantly differ from variability of HC participants. Based on previous research it might be speculated (29, 42), that an increase in OT in response to an attachment related stimulus is associated with seeking and finding of an internalized positive attachment representation. Furthermore, animal research has shown that the administration of morphine potently inhibits the secretion of OT and depresses the OT-sensitivity of the mammary gland, due to inhibition of the firing of supraoptic OT-neurons (63–66). Considering potential ceiling effects of methadone on the endogenous OT-system, its chronic administration could cause a maximum release of OT, so that further increases in OT are diminished, regardless of whether the person is triggered with an attachment related stimulus or not. Regarding the statistical tendencies observed in our sample which hints in the direction described above, more data is needed to further evaluate this line of interpretation.

Contradicting recent literature (15, 67), no significant differences between PUD patients and HC were found regarding adult attachment attitude using the AAS measure. Nevertheless, the non-significant associations showed there may be important relationships here which the current study was underpowered to detect and are consistent with the pattern observed in previous research (14, 67–69).

In general, the main results in this study may be influenced by several effects brought about by a combination of psychopharmacology, maintenance, and long-term psychotherapeutic treatment.

In addition, our findings designate a negative relationship between baseline OT-level and Comfort with Closeness in PUD patients. Corresponding to recent findings by Torres et al. (70), which suggested a negative correlation between the dose of maintenance therapy and Closeness as well as decreased Anxiety in patients undergoing maintenance therapy. Therefore, the mechanism of maintenance therapy might operate on the surface but helps PUD patients only to a limited extent in the formation of healthy interpersonal relationships and positive attachment representations that can be relied on in times of distress (15, 21).

Moreover, we observed tentative hints toward a link between OT-reactivity and increased Comfort with closeness which, however, did not achieve statistical significance. Similarly, Krause et al. (42) did not find significant associations between attachment security and OT-reactivity in lactating mothers. Hence, while a relationship between attachment and OT-reactivity may be a reasonable premise, more research should be done to further analyse this subject matter.

### Limitations and Future Perspectives

Findings of the present study are mainly limited by the sample size, the exclusion of the female gender and the use of self-report measures. Furthermore, the measurement of OT is controversially discussed in literature (43, 71).

Furthermore, nicotine abstinence was not given in PUD patients prior to the investigation in this study, which might be seen as a characteristic of PUD patients in maintenance treatment. However, in line with previous research, nicotine abuse was not related to OT (72, 73). Moreover, due to the explorative nature of this study, no control condition was administered, which limits the interpretability of the effects of the AAP on OT-levels. This shortcoming needs to be addressed in future studies. What is more, a recent study by Fuchshuber et al. (74) indicated a medium effect size regarding the difference in attachment security comparing PUD and HC groups (74). With respect to the relatively small sample size employed in this study, future research addressing this subject might take this to an account regarding the estimation of the required sample size. Along, to gain a more complete understanding of the relationship between attachment, OT and maintenance treatment, the investigation of abstinent SUD patients who are not undergoing maintenance therapy is of interest for future studies. Finally, cortisol and vasopressin, both known for their close interrelatedness with OT, should be taken into account (29, 30, 75, 76).

## Conclusion

This study suggests that peripheral OT levels in poly-drug users undergoing maintenance treatment do not show significant differences regarding responsive to an attachment related stimulus delivered *via* the Adult Attachment projective task compared to HCs. The meaning of this finding is complicated by a number of confound in the PUD group related to both the pharmacological and psycho-social treatments they are receiving. The current findings which indicate non-significant tendencies however are an important preliminary finding which we hope will motivate more research using an experimental paradigm to further explore this hypothesis.

## Data Availability Statement

This article contains previously unpublished data. Datasets are available on request.

## Ethics Statement

This study was carried out in accordance with the recommendations of the ethics guidelines of the Karl Franzens University of Graz, Austria. The protocol was approved by the ethics committee of the Karl Franzens University of Graz, Austria. Written informed consent in accordance with the Declaration of Helsinki was given by all subjects.

## Author Contributions

JT, EW, and HU conceptualized the study. JT, AK, FT, AR, and collected the data. JT, AB, SS, BR, TP, and KL analyzed the data. JT and AB interpreted the AAP data. JT, MH-R, HU, and AL drafted and revised the manuscript. EW, H-PK, AB, MH-R, HU, JF, and AL critically reviewed the manuscript. All authors contributed to the article and approved the submitted version.

## Conflict of Interest

The authors declare that the research was conducted in the absence of any commercial or financial relationships that could be construed as a potential conflict of interest.

## References

[1] World Health Organization WHO Expert Committee on Addiction-Producing Drugs [meeting held in Geneva from 25 to 30 November 1963]: thirteenth report. World Health Organization (1964).

[2] Volkow ND Koob GF McLellan AT Neurobiologic advances from the brain disease model of addiction. N Engl J Med (2016) 374(4):363–71. 10.1056/NEJMra1511480 PMC6135257 26816013

[3] Zellner MR Watt DF Solms M Panksepp J Affective neuroscientific and neuropsychoanalytic approaches to two intractable psychiatric problems: why depression feels so bad and what addicts really want. Neurosci Biobehav Rev (2011) (2011) 35:2000–8. 10.1016/j.neubiorev.2011.01.003 21241736

[4] Weigl M Anzenberger J Busch M Horvath I Turscherl E Bericht zur Drogensituation 2015. Vienna: Gesundheit Osterreich GmbH (2015).

[5] EMCDDA Annual report 2009: The State of the Drugs Problem in Europe. EMCDDA: Lisbon (2009).

[6] Hedrich D Alves P Farrell M Stöver H Møller L Mayet S The effectiveness of opioid maintenance treatment in prison settings: a systematic review. Addiction (2012) 107(3):501–17. 10.1111/j.1360-0443.2011.03676.x 21955033

[7] Flores PJ Addiction as an attachment disorder: Implications for group therapy. Int J Group Psychother (2001) 51(1):63–81. 10.1521/ijgp.51.1.63.49730 11191596

[8] Khantzian EJ Self-regulation and self-medication factors in alcoholism and the addictions. Similarities and differences. *Recent developments in alcoholism: An official publication of the American Medical Society on Alcoholism*, *the Research Society on Alcoholism*, *and the National Council on Alcoholism*. (1990) 8:255–71. 2185521

[9] Bowlby J The making and breaking of affectional bonds: I. Aetiology and psychopathology in the light of attachment theory. Br J Psychiatry Title (1977) 130(3):201–10. 10.1192/bjp.130.5.421 843768

[10] Bowlby J A secure base: Clinical applications of attachment theory (collected papers). London: Tavistock (1988).

[11] Milch W Sahhar N Zur Bedeutung der Bindungstheorie für die Psychotherapie Erwachsener. Psychotherapie (2010) 15(1):44–55.

[12] Bretherton I Munholland KA Internal working models in attachment: A construct revisited. In: Handbook of Attachment: Theory, Research and Clinical application. New York: Guildord Publications (1999). p. 89–111.

[13] Thompson RA Early attachment and later development. In: Cassidy J Shaver PR, editors. Handbook of attachment: Theory, Research and clinicla applications. New York, New York: Guildord Press (1999). p. 265–86.

[14] Schindler A Thomasius R Sack PM Gemeinhardt B KÜStner U Eckert J Attachment and substance use disorders: A review of the literature and a study in drug dependent adolescents. Attachment Hum Dev (2005) 7(3):207–28. 10.1080/14616730500173918 16210236

[15] Schindler A Bröning S A review on attachment and adolescent substance abuse: empirical evidence and implications for prevention and treatment. Subst Abuse (2015) 36(3):304–13. 10.1080/08897077.2014.983586 25424652

[16] Fairbairn CE Briley DA Kang D Fraley RC Hankin BL Ariss T A meta-analysis of longitudinal associations between substance use and interpersonal attachment security. Psychol Bull (2018) 144(5):532. 10.1037/bul0000141 29494194 PMC5912983

[17] Mikulincer M Shaver PR An attachment perspective on psychopathology. World Psychiatry (2012) 11(1):11–5. 10.1016/j.wpsyc.2012.01.003 PMC3266769 22294997

[18] Fuchshuber J Hiebler-Ragger M Kresse A Kapfhammer HP Unterrainer HF The influence of attachment styles and personality organization on emotional functioning after childhood trauma. Front Psychiatry (2019) 10:643. 10.3389/fpsyt.2019.00643 31543844 PMC6739441

[19] Hiebler-Ragger M Unterrainer HF The Role of Attachment in Poly-Drug Use Disorder: An Overview of the Literature, Recent Findings and Clinical Implications. Front Psychiatry (2019) 10:579. 10.3389/fpsyt.2019.00579 31507461 PMC6720034

[20] Feldman R The neurobiology of human attachments. Trends Cognit Sci (2017) 21(2):80–99. 10.1016/j.tics.2016.11.007 28041836

[21] Fonagy P Gergely G Target M The parent–infant dyad and the construction of the subjective self. J Child Psychol Psychiatry (2007) 48(3-4):288–328. 10.1111/j.1469-7610.2007.01727.x 17355400

[22] Andari E Duhamel JR Zalla T Herbrecht E Leboyer M Sirigu A Promoting social behavior with oxytocin in high-functioning autism spectrum disorders. Proc Natl Acad Sci (2010) 107(9):4389–94. 10.1073/pnas.0910249107 PMC2840168 20160081

[23] Galbally M Lewis AJ IJzendoorn MV Permezel M The role of oxytocin in mother-infant relations: a systematic review of human studies. Harv Rev Psychiatry (2011) 19(1):1–14. 10.3109/10673229.2011.549771 21250892

[24] Guastella AJ Einfeld SL Gray KM Rinehart NJ Tonge BJ Lambert TJ Intranasal oxytocin improves emotion recognition for youth with autism spectrum disorders. Biol Psychiatry (2010) 67(7):692–4. 10.1016/j.biopsych.2009.09.020 19897177

[25] MacDonald K MacDonald TM The peptide that binds: a systematic review of oxytocin and its prosocial effects in humans. Harv Rev Psychiatry (2010) 18(1):1–21. 10.3109/10673220903523615 20047458

[26] Feldman R The neurobiology of mammalian parenting and the biosocial context of human caregiving. Horm Behav (2016) 77:3–17. 10.1016/j.yhbeh.2015.10.001 26453928

[27] Kosfeld M Heinrichs M Zak PJ Fischbacher U Fehr E Oxytocin increases trust in humans. Nature (2005) 435(7042):673–6. 10.1038/nature03701 15931222

[28] Krause AL Borchardt V Li M van Tol MJ Demenescu LR Strauss B Dismissing attachment characteristics dynamically modulate brain networks subserving social aversion. Front Hum Neurosci (2016) 10:627. 10.3389/fnhum.2016.00627 27014016 PMC4783398

[29] Pierrehumbert B Torrisi R Ansermet F Borghini A Halfon O Adult attachment representations predict cortisol and oxytocin responses to stress. Attach Hum Dev (2012) 14(5):453–76. 10.1080/14616734.2012.706394 22856618

[30] Tops M van Peer JM Korf J Individual differences in emotional expressivity predict oxytocin responses to cortisol administration: Relevance to breast cancer? Biol Psychol (2007) 75(2):119–23. 10.1016/j.biopsycho.2007.01.001 17267094

[31] Uvnäs-Moberg K The oxytocin factor: Tapping the hormone of calm, love, and healing. Perseus: Basel (2003).

[32] Jobst A Padberg F Mauer MC Daltrozzo T Bauriedl-Schmidt C Sabass L Lower oxytocin plasma levels in borderline patients with unresolved attachment representations. Front Hum Neurosci (2016) 10:125. 10.3389/fnhum.2016.00125 27064696 PMC4811864

[33] Bowen MT Neumann ID Rebalancing the addicted brain: oxytocin interference with the neural substrates of addiction. Trends Neurosci (2017) 40(12):691–708. 10.1016/j.tins.2017.10.003 29128108

[34] Kovács GL Sarnyai Z Szabó G Oxytocin and addiction: a review. Psychoneuroendocrinology (1998) 23(8):945–62. 10.1016/s0306-4530(98)00064-x 9924746

[35] McGregor IS Bowen MT Breaking the loop: oxytocin as a potential treatment for drug addiction. Horm Behav (2012) 61(3):331–9. 10.1016/j.yhbeh.2011.12.001 22198308

[36] Panksepp J Bernatzky G Emotional sounds and the brain: the neuro-affective foundations of musical appreciation. Behav Process (2002) 60(2):133–55. 10.1016/s0376-6357(02)00080-3 12426066

[37] Peters S Slattery DA Flor PJ Neumann ID Reber SO Differential effects of baclofen and oxytocin on the increased ethanol consumption following chronic psychosocial stress in mice. Addict Biol (2013) 18(1):66–77. 10.1111/adb.12001 23126471

[38] Love TM Oxytocin, motivation and the role of dopamine. Pharmacol Biochem Behav (2014) 119:49–60. 10.1016/j.pbb.2013.06.011 23850525 PMC3877159

[39] Young LJ Wang Z The neurobiology of pair bonding. Nat Neurosci (2004) 7(10):1048–54. 10.1038/nn1327 15452576

[40] Machin AJ Dunbar R II The brain opioid theory of social attachment: a review of the evidence. Behaviour (2011) 148(9-10):985–1025. 10.1163/000579511X596624

[41] Zanos P Georgiou P Weber C Robinson F Kouimtsidis C Niforooshan R Oxytocin and opioid addiction revisited: old drug, new applications. Br J Pharmacol (2018) 175(14):2809–24. 10.1111/bph.13757 PMC6016632 28378414

[42] Krause S Pokorny D Schury K Doyen-Waldecker C Hulbert AL Karabatsiakis A Effects of the adult attachment projective picture system on oxytocin and cortisol blood levels in mothers. Front Hum Neurosci (2016) 10:627. 10.3389/fnhum.2016.00627 28008313 PMC5143683

[43] McCullough ME Churchland PS Mendez AJ Problems with measuring peripheral oxytocin: can the data on oxytocin and human behavior be trusted? Neurosci Biobehav Rev (2013) 37(8):1485–92. 10.1016/j.neubiorev.2013.04.018 23665533

[44] Nave G Camerer C McCullough M Does oxytocin increase trust in humans? A critical review of research. Perspect Psychol Sci (2015) 10(6):772–89. 10.1177/1745691615600138 26581735

[45] Leng G Sabatier N Measuring ocytocin and casopressin: bioassays, immunoassays and random numbers. J Neuroendocrinol (2016) 28(10):1–13. 10.1111/jne.12413 PMC5096068 27467712

[46] World Health Organization The ICD-10 classification of mental and behavioural disorders: Diagnostic criteria for research. World Health Organization (1993).

[47] George C West ML The Adult Attachment Projective Picture System: attachment theory and assessment in adults. New York, NY: Guilford Press (2012).

[48] Buchheim A Erk S George C Kächele H Kircher T Martius P Neural correlates of attachment trauma in borderline personality disorder: a functional magnetic resonance imaging study. Psychiatry Res (2008) 163(3):223–35. 10.1016/j.pscychresns.2007.07.001 18635342

[49] Humeniuk R Ali R Babor TF Farrell M Formigoni ML Jittiwutikarn J Validation of the alcohol, smoking and substance involvement screening test (ASSIST). Addiction (2008) 103(6):1039–47. 10.1111/j.1360-0443.2007.02114.x 18373724

[50] Schütz CG Daamen M van Niekerk C Deutsche Übersetzung des WHO ASSIST Screening-Fragebogens. Sucht (2005) 51(5):265–71. 10.1024/2005.05.02

[51] Derogatis LR The Brief Symptom Inventory–18 (BSI-18): Administration, Scoring and Procedures Manual. Minneapolis, MN: National Computer Systems (2000).

[52] Franke GH Ankerhold A Haase M Jäger S Tögel C Ulrich C Der Einsatz des Brief Symptom Inventory 18 (BSI-18) bei Psychotherapiepatienten. Psychother Psychosom Med Psychol (2011) 61(02):82–6. 10.1055/s-0030-1270518 21337286

[53] Collins NL Read SJ Adult attachment, working models, and relationship quality in dating couples. J Pers Soc Psychol (1990) 58(4):644–63. 10.1037//0022-3514.58.4.644 14570079

[54] Schmidt S Strauß B Höger D Brähler E Die Adult Attachment Scale (AAS)-teststatistische Prüfung und Normierung der deutschen Version. Psychother Psychosom Med Psychol (2004) 54(09/10):375–82. 10.1055/s-2003-815000 15343479

[55] Bowlby J Attachment and Loss. Attachment. Vol. 1. New York: Basic Books (1969).

[56] Christensen JC Shiyanov PA Estepp JR Schlager JJ Lack of association between human plasma oxytocin and interpersonal trust in a prisoner’s dilemma paradigm. PloS One (2014) 9(12):e116172. 10.1371/journal.pone.0116172 25549255 PMC4280178

[57] Nakagawa S A farewell to Bonferroni: the problems of low statistical power and publication bias. Behav Ecol (2004) 15(6):1044–5. 10.1093/beheco/arh107

[58] Perneger TV What’s wrong with Bonferroni adjustments. Bmj (1998) 316(7139):1236–8. 10.1136/bmj.316.7139.1236 PMC1112991 9553006

[59] De Leon G The therapeutic community: Theory, model, and method. New York, NY: Springer Publishing Company (2000).

[60] Crockford C Deschner T Ziegler TE Wittig RM Endogenous peripheral oxytocin measures can give insight into the dynamics of social relationships: a review. Front Behav Neurosci (2014) 8:68. 10.3389/fnbeh.2014.00068 24672442 PMC3949137

[61] Keating C Dawood T Barton DA Lambert GW Tilbrook AJ Effects of selective serotonin reuptake inhibitor treatment on plasma oxytocin and cortisol in major depressive disorder. BMC Psychiatry (2013) 13(1):124. 10.1186/1471-244X-13-124 23627666 PMC3643878

[62] Gołyszny M Obuchowicz E Are neuropeptides relevant for the mechanism of action of SSRIs? Neuropeptides (2019) 75:1. 10.1016/j.npep.2019.02.002 30824124

[63] Grell S Christensen JD Fjalland B The influence of morphine and naloxone on plasma oxytocin. Pharmacol Toxicol (1988) 63:274–6. 10.1111/j.1600-0773.1988.tb00953.x 2848232

[64] Evans RG Olley JE Rice GE Abrahams JM μ-and k-opiate receptor agonists reduce plasma neurohypophysial hormone concentrations in water-depriced and normally hydrated rats. Clin Exp Pharmacol Physiol (1989) 16(3):191–7. 10.1111/j.1440-1681.1989.tb01544.x 2541954

[65] Pumford KM Leng G Russell JA Morphine actions on supraoptic oxytocin neurones in anaesthetized rats: tolerance after icv morphine infusion. J Physiol (1991) 440(1):437–54. 10.1113/jphysiol.1991.sp018717 PMC1180161 1804971

[66] Russell JA Coombes JE Leng G Bicknell RJ Morphine tolerance and inhibition of oxytocin secretion by kappa-opioids acting on the rat neurohypophysis. J Physiol (1993) 469(1):365–86. 10.1113/jphysiol.1993.sp019818 PMC1143875 8271202

[67] Hiebler-Ragger M Unterrainer HF Rinner A Kapfhammer HP Insecure attachment styles and increased borderline personality organization in substance use disorders. Psychopathology (2016b) 49(5):341–4. 10.1159/000448177 27631792

[68] Jordan S Sack PM Thomasius MSM Küstner U Riedesser P 8 Schutz-und Risikofaktoren. In: Suchtstörungen im Kindes-und Jugendalter: das Handbuch: Grundlagen und Praxis. Stuttgart: Schattauer (2009). p. 127–38.

[69] Schindler A Thomasius R Petersen K Sack PM Heroin as an attachment substitute? Differences in attachment representations between opioid, ecstasy and cannabis abusers. Attach Hum Dev (2009) 11(3):307–30. 10.1080/14616730902815009 19455456

[70] Torres N Oliveira D Dias F Shaver P Panksepp J Testing a neuro-evolutionary theory of social bonds and addiction. Poster session at the Neuroscience of Affect, Attachment and Social Cognition Conference, Imperial College, London. (2013).

[71] Robinson KJ Hazon N Lonergan M Pomeroy PP Validation of an enzyme-linked immunoassay (ELISA) for plasma oxytocin in a novel mammal species reveals potential errors induced by sampling procedure. J Neurosci Methods (2014) 226:73–9. 10.1016/j.jneumeth.2014.01.019 24485867

[72] Chiodera P Volpi R Capretti L Bocchi R Caffarri G Marcato A Gamma-aminobutyric acid mediation of the inhibitory effect of endogenous opioids on the arginine vasopressin and oxytocin responses to nicotine from cigarette smoking. Metabolism (1993) 42(6):762–5. 10.1016/0026-0495(93)90246-k 8510522

[73] Seckl JR Johnson M Shakespear C Ughtman S Endogenous opioids inhibit oxytocin release during nicotine-stimulated secretion of vasopressin in man. Clin Endocrinol (Oxf) (1988) 28(5):509–14. 10.1111/j.1365-2265.1988.tb03685.x 3214943

[74] Fuchshuber J Unterrainer HF Hiebler-Ragger M Koschutnig K Papousek I Weiss E Pinpointing neural correlates of attachment in poly-drug use: A Diffusion Tensor Imaging study. Front Neurosci (2020) 14:596. 10.3389/fnins.2020.00596 32595448 PMC7300178

[75] Gordon I Zagoory-Sharon O Schneiderman I Leckman JF Weller A Feldman R Oxytocin and cortisol in romantically unattached young adults: associations with bonding and psychological distress. Psychophysiology (2008) 45(3):349–52. 10.1111/j.1469-8986.2008.00649.x 18266803

[76] Torres N Martins D Santos AJ Prata D Veríssimo M How do hypothalamic nonapeptides shape youth’s sociality? a systematic review on oxytocin, vasopressin and human socio-emotional development. Neurosci Biobehav Rev (2018) 90:309–31. 10.1016/j.neubiorev.2018.05.004 29738796

